# Enhancement of the Conductivity and Uniformity of Silver Nanowire Flexible Transparent Conductive Films by Femtosecond Laser-Induced Nanowelding

**DOI:** 10.3390/nano9050673

**Published:** 2019-05-01

**Authors:** Youwang Hu, Chang Liang, Xiaoyan Sun, Jianfen Zheng, Ji’an Duan, Xuye Zhuang

**Affiliations:** 1 The State Key Laboratory of High Performance Complex Manufacturing, College of Mechanical and Electrical Engineering, Central South University, Changsha 410083, China; huyw@csu.edu.cn (Y.H.); liangchangm@163.com (C.L.); m17375895997@163.com (J.Z.); duanjian@csu.edu.cn (J.D.); 2 East China Institute of Photo-Electronic IC, Bengbu 233033, China; zxye8888@163.com

**Keywords:** silver nanowire, transparent conductive films, welding, femtosecond laser, uniformity

## Abstract

In order to improve the performance of silver nanowire (AgNW) flexible transparent conductive films (FTCFs), including the conductivity, uniformity, and reliability, the welding of high repetition rate femtosecond (fs) laser is applied in this work. Fs laser irradiation can produce local enhancement of electric field, which induce melting at the gap of the AgNWs and enhance electrical conductivity of nanowire networks. The overall resistivity of the laser-welded AgNW FTCFs reduced significantly and the transparency changed slightly. Meanwhile, PET substrates were not damaged during the laser welding procedure in particular parameters. The AgNW FTCFs can achieve a nonuniformity factor of the sheet resistance as 4.6% at an average sheet resistance of 16.1 Ω/sq and transmittance of 91%. The laser-welded AgNW FTCFs also exhibited excellent reliability against mechanical bending over 10,000 cycles. The welding process may open up a new approach for improvement of FTCFs photoelectric property and can be applied in the fabrication of silver nanostructures for flexible optoelectronic and integration of functional devices.

## 1. Introduction

Flexible transparent conducting films (FTCFs) are widely used in flexible optoelectronic devices, such as flexible touch screens, flexible displays, transparent heaters, and solar cells [1,2,3,4]. Nowadays, the commercial FTCFs are mainly the indium-doped tin oxide (ITO) coated on polyethylene terephthalate (PET) substrate; however, ITO has its limitation of brittleness and the scarcity of indium, which may severely affect its applicability in flexible electronic devices [5,6]. To keep up with market demand, several new FTCFs, such as graphene, carbon nanotubes, and metallic nanostructures are widely researched to replace ITO [7,8,9].

Among these candidate electrode materials, silver nanowires (AgNWs) shows well promising, mainly due to its excellent conductivity, high optical transmittance and mechanical flexibility. However, the conductivity, uniformity, and reliability of nanowire network are determined by the resistance of wire-to-wire junction. Additionally, these characteristics restrict the application of AgNW FTCFs [10]. For example, the standard deviation of sheet resistance of touch panels should be lower than 10% to precisely locate the touching coordinate [11].

Recently, some methods have been reported to enhance the wire-to-wire junction conductivity, such as mechanical pressing [12], thermal annealing [13], plasmonic optical welding [14,15], and chemical treatment [16]. While the mechanical pressing could achieve good smoothness and low sheet resistance of metallic nanowires films, the sheet resistance uniformity cannot satisfy the requirement of high-end applications. The thermal treatment with a hot plate was prone to break up of the nanowires. Plasmonic optical welding can generate hot spots only at the nanowire junctions to the formation of a fully welded metal nanowire network. However, the process caused surface damage of flexible substrates easily. As to the chemical treatment, the chemical residuals decreased optical transmittance. Thus, a more effective post-treatment method to improve the comprehensive performance of AgNW FTCFs is urgently needed [17].

Recently, laser welding has been applied in the welding of AgNWs [18,19]. It is a non-contact and non-contaminate technique. The principle of this method is excitation of surface plasmon resonances (SPRs) in and around nanomaterials, which can lead to the enhancement of the electrical field in the nanogap between non-contacting nanomaterials and be followed by electrical field induced nanomaterial melting, resulting in the migration of material into the gap [18]. Previous studies on nanowelding of metallic nanowires mainly used nanosecond and longer pulsed lasers. However, the thermal effect of nanosecond laser was obvious, and it can be used to induce melting of nanowires for the assembly of spatially correlated nanoparticles [20,21]. Spechler et al. [22] showed the nanosecond laser processing damaged the electrode by melting the AgNWs into conglomerations. Ha et al. [23] indicated nanosecond laser irradiation inducing more damage to the PET substrate than femtosecond laser irradiation. That is, because the thermal diffusion length of the sample is larger during the nanosecond laser irradiation.

Femtosecond (fs) laser with ultrashort pulse durations and high peak power without causing thermal damage to the part is usually employed in nanostructures manufacture and nanomaterials modification [24,25,26,27]. It is an ideal tool for the excitation of surface plasmon resonances (SPRs), because the fs laser excitation can minimize thermal damage in the adjacent nanomaterials and substrate materials [28,29]. The interconnection of individual nanoparticles under strong optical field excitation to form joint in the absence of filler material has been reported. For example, Huang et al. [30] produced a metallic interconnection between two adjacent silver submicron/nanoparticles by using low fluence fs laser irradiation. Lin et al. [31] reported the in situ joining of AgNWs without additional filler material by controlled irradiation of fs laser. Ha et al. [23] welded the silver nanowire networks using ultrashort laser pulses to minimize thermal damage of polymer substrate. And, the sheet resistance and transmittance of the AgNW network were improved. These researches demonstrate that fs laser radiation can be used to generate filler-free joint between nanomaterials to form nanowelding. However, there are rarely research focusing on the uniformity and reliability of AgNW FTCFs.

In this study, we report a method of fs laser welding to improve the conductivity, uniformity and reliability of AgNW FTCFs without substrate damage. In order to avoid nanowire fusing, we chose the high repetition rate fs laser with the single pulse energy at nJ magnitude, and simulate the local field enhancement induced by perpendicular nanowires. The defocus processing method is adopted to improve efficiency and uniformity. The AgNW FTCFs with high electrical conductivity and uniformity has been achieved. The experiments of bending, local thermal deformation of substrate, and removal of welded nanowires have been carried out to study the reliability of the welded AgNW FTCFs.

## 2. Material and Methods

### 2.1. Material and Film Coating

AgNWs suspension used in this work was purchased from Haoxi Nanomaterials (HAOXI Inc., Shanghai, China) with average length of 16 µm and diameter of 60 nm. To fabricate FTCFs, The AgNW suspension was homogenized by supersonic vibration for 2 min. The AgNWs were deposited on the clean PET substrates using spin-coating at 1800 rpm for 60 s.

### 2.2. Laser Welding AgNW FTCFs

**Figure 1:**
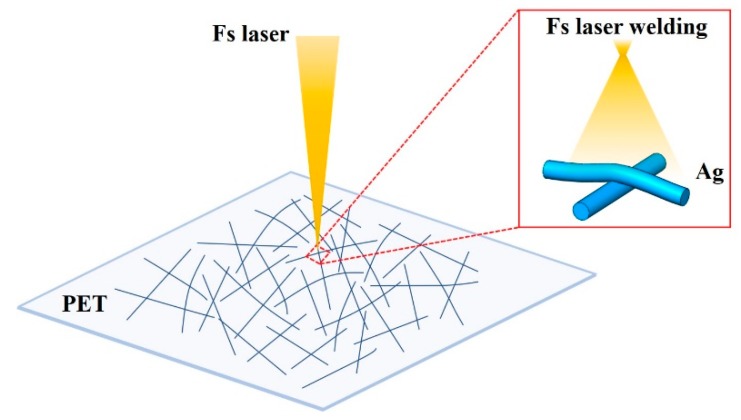
Schematic illustration of fs laser welding of AgNWs on PET film.

Welding of the simples exposed to the fs laser irradiation at the fluence of approximately 0.2~2.0 μJ/cm^2^. The fs laser welding procedure for samples are schematically illustrated in Figure 1. The fs laser (Spectra Physics) with a central wavelength 800 nm, a pulse duration 120 fs and a repetition rate 80 MHz were used in this work. The maximum power of fs laser was 1.4 W and the irradiation energy was attenuated by a half wave plate and a Glan prism. The fs laser source had a Gaussian intensity profile that tightly focused on the top surface of the target material through a lens of focal length 50 mm. The sample was placed perpendicular to the laser beam at 1 mm after focal point, and the laser spot size was about 800 μm. A large area welding was achieved by scanning the laser spot on the sample through controlled movement of X-Y stage. The sample moved in the x direction with a constant scanning speed. After each scan, the sample was moved in the y direction by a distance of 500 μm. The scanning speed was in the range of 0.5 mm/s to 5 mm/s.

### 2.3. Performance Characterization

The morphology of the AgNWs was analyzed using SEM (SEM, TESCAN, Brno, Czech). Crystallographic properties of AgNWs were characterized by X-ray diffraction (XRD, Max 2500, Rigaku, Tokyo, Japan) The sheet resistance of the specimen before and after laser scanning were measured by the four-point probe system (SDY-4D, Guangzhou, China). The transmittance and reflection spectra were collected by an UV-VIS spectrophotometer equipped with an integrating sphere. (Cary 5000, Varian, Palo Alto, CA, USA). The value of haze was determined as the degree of diffuse light scattering through the films. It was calculated according to the relationship Haze = (*T_tot_* − *T_spec_*)/*T_tot_*, where *T_tot_* is the total transmittance and *T_spec_* is the specular transmittance of the films. Stretching were conducted by applying mechanical loads to the sample using a computer-controlled automatic stand (Suns, Shenzhen, China). The stability tests of the samples were stored in an environmental test chamber up to 30 days (Excal 2214, Climats, France), where the relative humidity and the temperature were 85% RH and 85 °C, respectively. Simulations were carried out using the finite element method (COMSOL Multiphysics 5.3a). In the simulation, the AgNWs are 60 nm in diameter, 2 µm in length, and overlap at a single point.

## 3. Results and Discussion

### 3.1. Morphology of the Laser-Welded AgNWs

**Figure 2:**
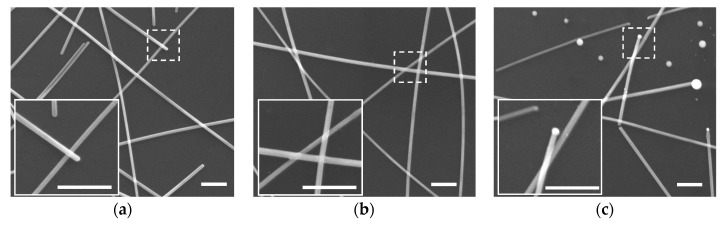
SEM images of the AgNWs on the silicon substrate (**a**) before fs laser irradiation, after fs laser irradiation at fluence of (**b**) 1.2 μJ/cm^2^ and (**c**) 2.0 μJ/cm^2^. Scale bar is 500 nm.

Figure 2 shows the micrographs of AgNWs networks with different laser fluences. Figure 2a is the unprocessed sample, which displays the randomly arranged AgNWs and the AgNWs simply establish physical contact with one another on the silicon substrate. When the AgNWs treatment at laser fluence of 1.2 μJ/cm^2^, the morphology of junctions began to slightly melt and weld together. Meanwhile, the nanowires surrounding each junction were unaffected and no further morphological change was observed (Figure 2b). Further increase the laser fluence substantially resulted in damage of the AgNWs (Figure 2c). The balling of silver appeared at the ends of nanowires and some nanowires separated into several nanoparticles. However, the central part of most AgNWs still remained in the original morphology. This result is different from that observed for heating with a hot plate. The thermal treatment of hot plate appeared as an undesired breaking up of the nanowires aligned with a Rayleigh instability [32]. These differences highlight the excellence of the fs laser nanowelding method, which allows junctions in the nanowire network to be welded and electrically connected without breaking up the wires.

### 3.2. Numerical Analysis of the Electric Field Intensity for Laser-Welded the AgNWs

**Figure 3:**
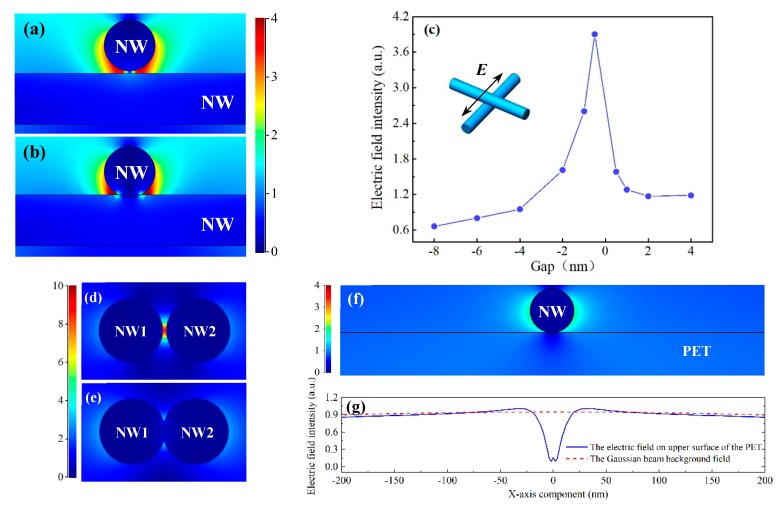
Simulated cross-sectional electric field distribution in the adjacent of two perpendicularly crossed AgNWs at the gap of (**a**) 2 nm and (**b**) −2 nm, respectively. (**c**) Field intensity in the intersection area at different gap distances calculated for the structures in the inset. The polarized direction of laser is perpendicular to the top nanowires (black arrow). Negative values on the *x* axis correspond to interpenetrating nanowires. Simulated electric field distribution between a pair of parallel AgNWs with at the gap of (**d**) 2 nm and (**e**) −2 nm, respectively. (**f**) Electric field distribution between nanowire and PET surface. (**g**) Line plot of the x component of the electric field (blue line) on upper surface of the PET and the Gaussian beam background field (red line).

In order to examine the field localization with subwavelength-sized AgNWs, we calculated the electromagnetic field distribution between two vertical nanowires. In the side view between the two nanowires, as shown in Figure 3a,b, we see the two lobes of highly concentrated field intensity and this predicted up to a 4-fold enhancement in the field intensity at the junction of the AgNWs. The melting of that area would greatly increase the contact area between the two nanowires. At high intensity optical excitation, the effect of field enhancement at the gap between AgNWs is magnified on account of enhanced surface plasmon coupling arising from the presence of nanowire within the optical near field [18,33,34]. The electromagnetic wave can generate thermal heating that is proportional to the electric field intensity closer to the target material, and induce melting at the gap of the AgNWs. Figure 3c plots the field intensity in the intersection area at different gap distances. It displays that the effect of field intensity increases with the AgNWs come closer together. Once the nanowires touch, the local electric filed enhancement effect declines rapidly and heating becomes less effective and then the AgNWs cools. This phenomenon illustrates that laser nanowelding has the self-limiting property. Similar to crossed junctions, the field intensity between a pair of aligned AgNWs is also extremely sensitive to the interwire gap size (Figure 3d,e). With the same gap size of 2 nm, the simulation results indicated that the maximum local electric field intensity at the parallel interstitial single pair AgNWs is bigger than at the parallel interstitial. Figure 3f shows electric field distribution between nanowires and PET surface. The electric field strength on both sides of the nanowire (blue line) is higher than that of the Gaussian beam background field (red line), as shown in Figure 3g. This will cause the PET around the nanowires to melt more easily.

### 3.3. Sheet Resistance and Transmittance

**Figure 4:**
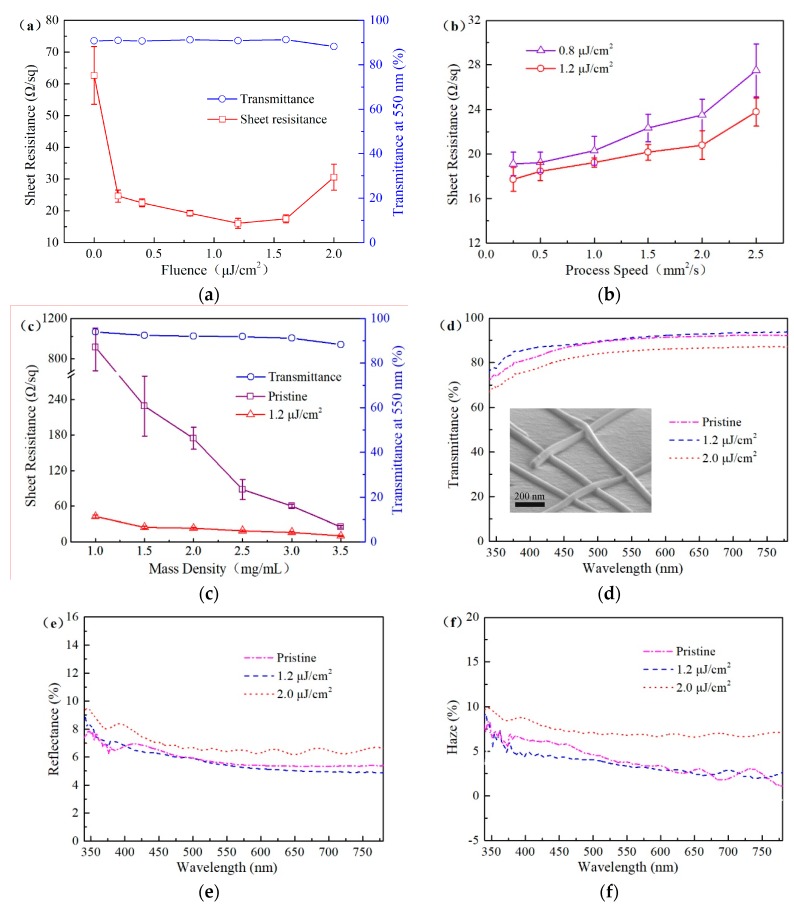
Change in the sheet resistance and transmittance of AgNW FTCFs at (**a**) different laser fluences and (**b**) process speeds. (**c**) The sheet resistance and transmittance of the AgNW FTCFs at various mass density of AgNWs solution and following laser welding. (**d**) Transmittance, (**e**) reflectance, and (**f**) haze of AgNW FTCFs before and after laser welding. Inset: The SEM images of AgNWs spined-coating onto PET film for laser-welded sample.

Owing to the weak contact between nanowires and large contact resistance, the prepared AgNW FTCFs exhibit poor electrical conductivity. The laser welding process is subsequently applied on the prepared AgNW FTCFs to improve the electrical conductivity. Figure 4a shows the sheet resistance and transmittance of the AgNW FTCFs at various fluences of 0.2~2.0 μJ/cm^2^ and fixed process speed at 1 mm^2^/s. Each sample was measured 10 times at random positions and the error bars were calculated, as shown in Figure 4. The sheet resistance and transmittance sample before laser irradiation is 62.7 Ω/sq and 91% at 550 nm, respectively. After fs laser welding, the whole resistance of the AgNW FTCFs is rapidly decreased, and the eventual sheet resistance could be as low as 16.1 Ω/sq. Additionally, the transmittance change of AgNW FTCFs are small, which shows that the fs laser direct-write method is directly applied well to welding AgNW FTCFs. The sheet resistance of the AgNW FTCFs at various process speeds are shown in Figure 4b. Fixing laser fluence at 0.8 or 1.2 μJ/cm^2^, the sheet resistance increased with the increasing of the process speed.

For fabricating the FTCFs with high optical transmittance and conductivity, the optical transmittance and sheet resistance should be taken into account simultaneously because these parameters generally counteract with each other. Figure 4c shows the eventual sheet resistance of the laser-welded AgNW FTCFs formed from various mass density of AgNWs solution scanned by the fs laser with fluence of 1.2 μJ/cm^2^ and process speed of 1 mm^2^/s. It is distinct that the sheet resistance decreases dramatically through increasing the mass density of AgNWs solution. But the transmittance falls from 92.7% to 88.2%. At the mass density of 3 mg/mL, the sample after laser welding presents a sheet resistance as 16.1 Ω/sq at a transmittance of 91%.

The transmittance, reflection and haze of the AgNW FTCFs over the visible wavelengths is shown in Figure 4b,d,e. Compared to prepared AgNW FTCFs, the transmittance, reflection, and haze keep the same or have minor change at laser fluence of 1.2 μJ/cm^2^. The inset of Figure 4d shows the micromorphology of AgNWs spined-coating onto PET film for laser-welded sample at 1.2 μJ/cm^2^, and no damage was found on the surface of the sample. When the laser fluence increased to 2.0 μJ/cm^2^, the transmittance decreased and the reflection increased significantly. This phenomenon can be attributed to excessive laser energy induced slight deformation of the PET substrate, which leads to a decrease in transmittance and an increase in reflection and haze.

**Figure 5:**
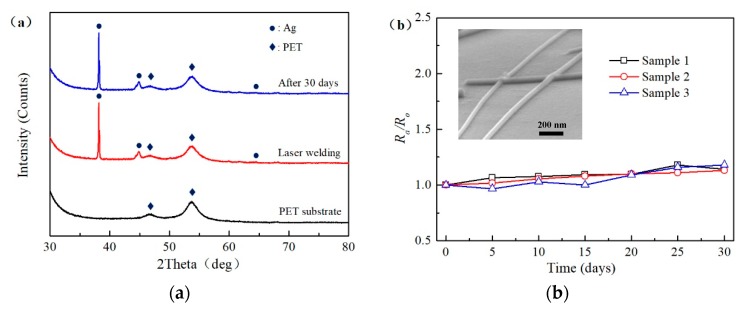
(**a**) XRD results of PET and laser-welded AgNW FTCFs. (**b**) Plot of the sheet resistance versus time for laser-welded AgNW FTCFs after exposure to humid and hot air (humidity: 85%, temperature: 85 °C) for 30 days. Inset: the morphology of nanowire after the end of the 30-days stability test. *R*_0_ is the initial resistance, *R_a_* is the resistance measured after the experiment.

Figure 5a shows the XRD results of PET substrate and laser-welded AgNW FTCFs. The two main diffraction peaks of PET substrate were measured before spin coating experiment (black line). After laser processed the AgNWs network, the peaks of Ag and PET were observed and the peaks of silver oxide (red line) was not detected. The blue line shows the XRD result of the laser-welded AgNWs network exposed to air environments for 30 days. The various peaks related to the chemical structures of Ag and PET still existed. This demonstrates that laser-welded AgNWs present a high oxidation resistance under the ambient environments. In order to test thermal oxidation stabilities of welded AgNW FTCFs, the samples were exposed to the condition at 85 °C and 85% RH for 30 days. Figure 5b shows the sheet resistance of the AgNW FTCFs are almost unchanged. This confirms that the conductivity of the AgNWs network was not destroyed after the thermal oxidation stability test. The inset of Figure 5b shows the morphology of nanowire after the end of the 30-days stability test. The nanowires remained a normal random network structure with sharp wires, only a small amount of irregular particles appeared on the surface. Therefore, it is possible to format amorphous Ag oxide.

### 3.4. Distribution of Sheet Resistance of the AgNW FTCFs

The initial sheet resistance value of the AgNW FTCFs present a wider range of ~45 to ~1000 Ω/sq and large resistance also corresponds to large standard deviation, as shown in Figure 4c. The uniformity of the AgNW sheet resistance is determined by a point contact electrical path [35,36]. In this work, we found that the laser welding played an important role in enhancing the resistance uniformity of AgNW FTCFs. The effect of fs laser welding on the sheet resistance uniformity was investigated by changing the mass density of AgNWs solution. In order to evaluate the sheet resistance uniformity of the samples, relative evaluation standard (RSTD) was introduced. The value of RSTD was determined as the deviation of the sheet resistance of the sample away from the average value. The RSTD was expressed by dividing the total standard deviation by the average of the sample [11]. The RSTD can be calculated as

$$\mathrm{RSTD}=\sqrt{\frac{1}{n}\frac{\sum_{i=1}^{n}{\left(R_{i}-\bar{R}\right)}^{2}}{{\bar{R}}^{2}}}$$

where *n* is the number of measurements on the sample of different position, *R_i_* is the measured resistance and $\bar{R}$ is the average resistance of all the measuring points. The smaller the value of RSTD shows the more uniform resistance of the film.

**Figure 6:**
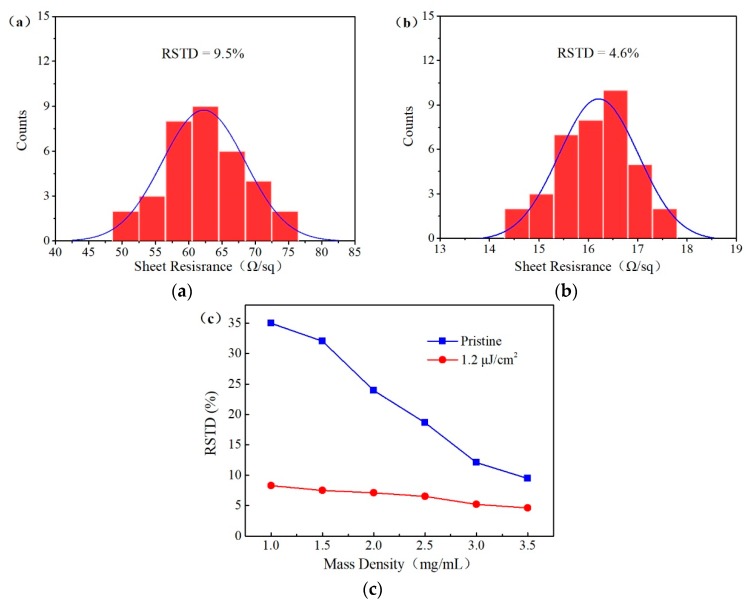
Distribution of sheet resistance of AgNW FTCFs (**a**) befor and (**b**) after laser welding. (**c**) The RSTD of the sheet resistance for AgNW FTCFs with different mass density of AgNWs.

The RSTD of AgNW FTCFs before and after laser welding are shown in Figure 6 at mass density of 3 mg/mL. The FTCFs were divided into 36 parts of the same size and the sheet resistance of each part was measured. The RSTD of the sheet resistance was 4.6% after laser welding at fluence of 1.2 μJ/cm^2^, much lower than that of the film before laser irradiation (9.5%), as shown in Figure 6a,b. Figure 6c shows the RSTD of the AgNW FTCFs at different mass density of AgNWs solution and following laser welding. Before laser irradiation, it is distinct that the RSTD of AgNW FTCFs increases monotonously with the mass density of AgNWs reducing. Compared to the before laser irradiation, the RSTD of all AgNW FTCFs after laser welding are dramatically decreased. Especially when the mass density of AgNWs solution is small, the change of RSTD is large. Finally, the RSTD decreases to a minimum value of 3.8% at a mass density of 3.5 mg/mL.

### 3.5. Reliability of the AgNW FTCFs

**Figure 7:**
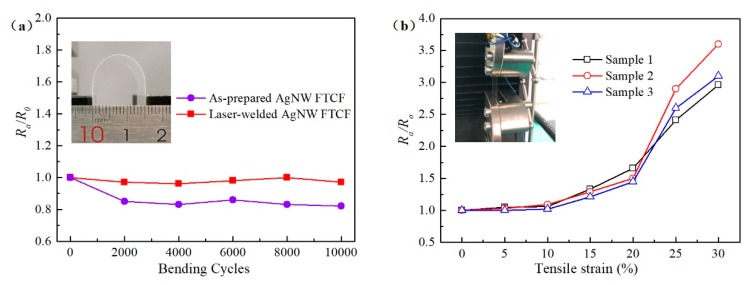
(**a**) The results of cyclic bending test of the AgNW FTCFs. (**b**) The results of stretching test of the AgNW FTCFs after laser welding. Insets: digital images of the samples being tested.

The reliability of the AgNW FTCFs was further investigated by bending test after laser welding at fluence of 1.2 μJ/cm^2^. The results reveal that the sheet resistance of the laser-welded AgNW FTCFs shows less than 3% deviation after 10,000 cycles with a bending radius of 15 mm, while the sheet resistance of as-prepared AgNW FTCFs shows decreasing and eventually down to a steady ratio at the same bending condition, as shown in Figure 7a. For as-prepared samples, the stretch may induce cold-welding at overlapping nanowires during the bending test, which caused the sheet resistance of as-prepared AgNW FTCFs decreasing. A small change in the sheet resistance of the laser-welded AgNW FTCFs indicates that the welding joints have achieved a strong connection after laser welding. To test the sheet resistance and stretchability of the AgNW FTCFs after laser welding, the stretching tests were carried out. As schematically illustrated in Figure 7b, the samples were tested on a stretcher with the stretching direction parallel to the longitudinal axis of the samples. When the tensile strain is ≤10%, the resistance has only a slight change. After the tensile strain is greater than 10%, the surface resistance of the film increases rapidly. This may be due to the breakage of partial AgNWs after experiencing high strain (≥15%), which destroys the conductivity of the AgNWs network.

**Figure 8:**
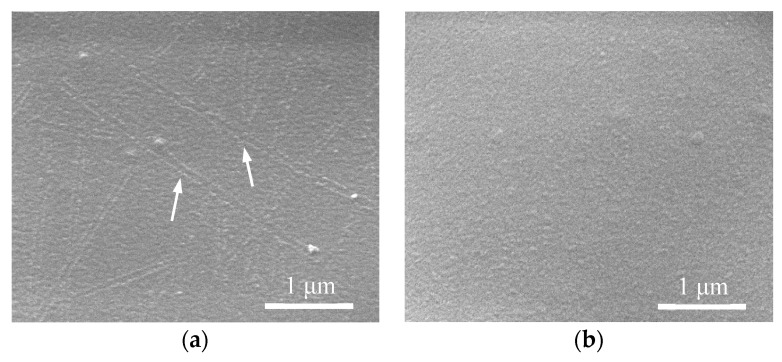
SEM image of PET surface after ultrasonic cleaning (**a**) the pristine AgNWs FTCFs and (**b**) laser-welded AgNWs FTCFs, respectively.

In order to verify the bonding properties between nanowires and PET surface, we removed the pristine and laser processed AgNWs on PET by ultrasonic cleaning. Figure 8a shows the surface of the PET before laser welding shows clear PET surface without local melting region. However, the PET surface after laser processed appeared local melted along the AgNWs (groove pointed by white arrow) due to the local high temperature, as shown in Figure 8b. That is because the simulation results show that the local electric field enhancement occurred at the interface between nanowire and PET. Surface plasmon polaritons of AgNWs was excited, resulting in local high temperature [14,37]. This caused embedment of AgNWs into PET and increased contact area between nanowires and substrate. The adhesion between nanowires and PET surface was enhanced. The melting point of PET is about 240 °C [38,39]. Thus, we estimate the temperature of the nanowire to be about 240 °C during the laser irradiation. This result is in reasonable agreement with the result of the simulation by Jung et al. [14] Additionally, in spite of intensive laser processing on thermally sensitive PET, the film deformation or other negative side effects on PET properties were not observed.

**Figure 9:**
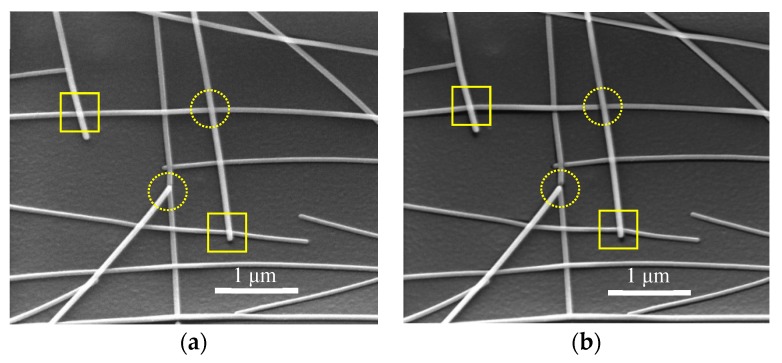
SEM images of the AgNW FTCFs on the PET substrate. (**a**) Irradiating for the first time. (**b**) Irradiating for the second time.

Figure 9 shows a set of SEM images of laser-welded AgNWs network area with different irradiation time of electron beam during the SEM measurement. Experienced the first time of electron irradiation, the solid rectangles and dotted circles indicate being connected, but did not fracture nanowires (Figure 9a). However, experienced the second time of electron irradiation, the PET substrate occurred deformation that because the continuous action of electron beam produced thermal effects. The deformation of PET substrate generated tensile force, which may break the laser-welded AgNWs. As shown in dotted circles of Figure 9b, the fractured nanowires are ruptured at the junction point, but welding spots of wire-wire junction are not destroyed. This suggests again that fs laser is an effective method for the welding of AgNWs and formatted stabilized nanowelding. Some junction points shown in Figure 9 are welded and the other points have no obvious weld morphology. This is because the distance between nanowires on each cross is random after spin coating. The field strength between nanowires depends on the distance between nanowires. With the increasing of nanowires spacing the field strength is getting weaker. The different spacing between the nanowires randomly distributed on the surface of the film caused the different welding field strength. Once the laser intensity is too high, the AgNWs would be burnt. In order to avoid the burning of nanowires, optimized parameter only can weld partial of the cross evidently. In addition, the fs laser direct-write method does not damage of the PET substrate in specific process parameters, as shown in Figure 9. This indicates that the fs laser welding process is also compatible with flexible PET substrates on account of the reduced heat-affected zone of the focused spot.

## 4. Conclusions

In summary, the conductivity and uniformity of the AgNW FTCFs were improved using a fs laser welding method. Fs laser irradiation generates local heat at the junctions between the nanowires due to the field enhancement, which produced melting at the gap of the AgNWs. Through this process, the overall resistance of the AgNW FTCFs reduced significantly and the transparency of FTCFs changed slightly. Meanwhile, PET substrates were not significantly damaged during the laser welding procedure in particular parameters. The AgNW FTCFs can achieve a nonuniformity factor of the sheet resistance as 4.6% at an average sheet resistance of 16.1 Ω/sq and transmittance of 91%. The laser-welded AgNW FTCFs also exhibited excellent reliability against mechanical bending over 10,000 cycles and showed the lifetime of 30 days under an atmosphere at 85 °C and 85% RH. The numerical analysis of the electric field intensity confirmed that welding of the AgNWs be incited by 800 nm laser. Considering that the welding process fabricated of AgNW FTCFs with excellent photoelectric property and eliminates the effect on the substrate, this approach would offer a new route to fabrication of flexible transparent electrodes.
